# Young-onset stroke in the UAE: A single-centre retrospective analysis of risk factors and outcomes

**DOI:** 10.1371/journal.pone.0355300

**Published:** 2026-08-06

**Authors:** Sobia Siddiq, Saryia Adra, Kamel A. Samara, Batool Aldaher, Mohamad Emad Aldeen Mahfouz, Nour Sakan, Fathima Musfira, Roomiyah Riyaz Assadi, Ahlam Mohamed Almarzooqi, Hiba Jawdat Barqawi, Eman Abu-Gharbieh

**Affiliations:** 1 Princess Royal Hospital, University hospital Sussex, NHS foundation, RH16 4EX Haywards Heath, United Kingdom; 2 Department of Rheumatology, Al Qassimi Hospital, Emirates Health Services, Sharjah, United Arab Emirates; 3 Department of Medicine, NYC Health + Hospitals/Harlem, New York, New York, United States of America; 4 College of Medicine, University of Sharjah, Sharjah, United Arab Emirates; 5 Jefferson Abington Hospital, Abington, Pennsylvania, United States of America; 6 The University of Tennessee at Chattanooga, Chattanooga, Tennessee, United States of America; 7 HCA Healthcare, Centerpoint Medical Center, Independence, Missouri, United States of America; 8 Research Institute for Medical and Health Sciences, University of Sharjah, Sharjah, United Arab Emirates; 9 School of Pharmacy, The University of Jordan, Amman, Jordan; Foshan Sanshui District People's Hospital, CHINA

## Abstract

**Background:**

Young-onset stroke represents a growing public health concern, yet data from the United Arab Emirates (UAE) remain limited. This study aimed to examine the risk factors and clinical outcomes associated with young-onset stroke in the UAE.

**Methods:**

A retrospective chart review was conducted on 419 patients aged below 50 years who were admitted at Al Qassimi Hospital, Sharjah, UAE, with a diagnosis of stroke between 2016 and 2022. Data on demographics, comorbidities, stroke subtype, management, and outcomes were collected and analyzed using Python-based statistical libraries.

**Results:**

The study population comprised predominantly of male individuals (78.28%), with most patients originating from South Asia (63.96%) and the Middle East and North Africa region (19.33%). Ischemic stroke was the most common subtype (50.12%), followed by intracerebral hemorrhage (27.21%) and non-traumatic subarachnoid hemorrhage (22.67%). Hypertension (45.45%) and diabetes mellitus (24.42%) were the most prevalent comorbidities. Only 0.24% of patients underwent mechanical thrombectomy, and 9.41% received intravenous thrombolysis. The overall mortality rate was 12.68%, with coronary artery disease significantly associated with death (p < 0.001). South Asian patients were 2.19 times more likely to have large-artery atherosclerosis compared to non–South Asians (95% CI: 1.11–4.30, p = 0.02).

**Conclusions:**

Young-onset stroke in the UAE is characterized by a high burden of modifiable cardiovascular risk factors, particularly hypertension and diabetes mellitus. Targeted preventive strategies and region-specific research are essential to reduce disease burden and improve patient outcomes.

## Introduction

Despite rapid advancements in the prevention, diagnosis and treatment of stroke it remains the second leading cause of death and disability globally [1]. In 2019, approximately 12.2 million new stroke cases were reported worldwide marking a 70% increase since 1990 [1]. This surge was accompanied by an 85% increase in stroke prevalence during the same period [1]. In the Middle East and North Africa (MENA) region, over 800,000 new stroke cases were recorded in 2019 with a prevalence of approximately 7.7 million cases [1]. Additionally, the United Arab Emirates (UAE) had the highest age-standardized stroke point prevalence in the MENA region in 2019 [2].

While the rising stroke burden affects all demographics, young-onset stroke (defined as stroke occurring in individuals younger than 50 years of age [3]) particularly leads to profound socioeconomic consequences as it often disables individuals during their most productive years [4]. The underlying pathophysiology of young-onset stroke is not as well-established as stroke in older adults. Traditional risk factors such as undiagnosed thrombophilia, autoimmune diseases, patent foramen ovale, and arterial dissections are implicated in young-onset strokes [5]. However, young adults are also affected by factors such as smoking, oral contraceptive use, migraines with aura, trauma, illicit drug use, and pregnancy-related conditions [3]. These factors play a more significant role in younger populations compared to their older counterparts. Additionally, it is postulated that young-onset stroke is largely due to the increasing prevalence of modifiable risk factors such as obesity and diabetes mellitus [6].

The compounding effect of lifestyle-related aetiology is highly alarming for the UAE and the broader MENA region. Notably, the MENA region ranks among the highest globally for diabetes mellitus prevalence, with the UAE experiencing an 84.5% increase in diabetes mellitus prevalence rate between 1990 and 2021 [7]. Additionally, obesity and hypertension are highly prevalent in the UAE and pose a significant public health risk. It is estimated that 25% of the UAE population have diabetes mellitus and about one-third suffer from hypertension [8]. Furthermore, while published regional paediatric data is scarce, a recent review article highlighted that 17.35% of UAE school children were obese, with an upward trend over the years [9].

Driven by the metabolic risk factors epidemic, it is expected that the incidence of young-onset stroke will also increase [10]. A global study noted that the incidence of ischemic stroke in those aged 35–39 increased from 22.1 to 22.7 per 100,000 from 1990 to 2019 [4] underscoring the growing burden among younger populations. Aggressive primary and secondary prevention strategies targeting the modifiable risk factors are needed.

Despite the growing disease burden, data on the epidemiology and risk factors of young-onset stroke in the UAE is scarce which hinders effective public health interventions. Hence, this study aimed to evaluate the epidemiological characteristics, risk factor profiles, and clinical outcomes of young-onset stroke in the UAE. Ultimately, these findings seek to inform targeted prevention strategies aimed to reduce the burden of stroke on individuals and society.

## Methodology

### Study design and population

A retrospective chart review was conducted at Al Qassimi Hospital (AQH) in Sharjah, UAE. AQH, the largest governmental tertiary care facility in the Northern Emirates, serves a broad patient base with over 350 beds. The study included all patients admitted with stroke between January 1^st^, 2016, and December 31^st^, 2022, who met the inclusion criteria outlined below. An initial list of stroke cases was identified from the hospital’s electronic medical records using the International Classification of Diseases (ICD-10-CM) codes for stroke (Supplemental Table 1). Following the automated identification process, a team of five trained interns and residents manually reviewed the patients’ charts and extracted relevant data for variables not captured in the electronic medical records.

This study was conducted in accordance with the ethical standards of the Research Ethics Committee at the University of Sharjah (REC-23-01-23-01-F) and the Ministry of Health and Prevention Research Ethics Committee (MOHAP/DXB-REC/ J.J.J /No.74 / 2023) and with the 1964 Helsinki Declaration and its later amendments. Given the retrospective nature of the study, the requirement for informed consent was waived by both IRBs. Data collection was conducted between 10 July 2023 and 12 January 2024.

### Diagnostic protocol

At our centre, the standard protocol for all suspected acute stroke patients includes non-contrast CT brain, CT perfusion, and CT angiography (CTA) of the head and neck to evaluate for large vessel occlusion, stenosis, or dissection. As for cardiac evaluation, patients are monitored for a minimum of 24 hours on the telemetry floor, and a transthoracic echocardiogram (TTE) is performed prior to discharge. Advanced cardiac investigations, such as transoesophageal echocardiography or prolonged ambulatory Holter monitoring (>24 hours), are performed at the discretion of the treating physician based on individual risk factors. Implantable loop recorders are not utilized at the centre.

### Data collection

A comprehensive literature review was conducted to identify gaps in the existing knowledge regarding stroke epidemiology, management, and outcomes. Based on this review, a structured data collection spreadsheet was developed to facilitate consistent and thorough manual data entry.

The data collected encompassed a wide range of variables, including demographic information (age, sex, and region of origin), comorbid health conditions (hypertension, diabetes mellitus, hyperlipidaemia, coronary artery disease and atrial fibrillation), past medical history, and family history of stroke or cardiovascular diseases. Details of clinical assessment including vital signs (blood pressure, heart rate), BMI, and glucose levels on admission were also noted. Furthermore, details of imaging results, type of stroke (ischemic or hemorrhagic), hemorrhagic transformation within 24 hours, ECG changes, basic blood workup, treatment interventions (thrombolytic therapy, anticoagulation, or mechanical thrombectomy), and outcomes (including survival) were also documented. Stroke subtype (ischemic stroke, non-traumatic intracerebral hemorrhage and non-traumatic subarachnoid hemorrhage [SAH, to refer to non-traumatic SAH from hereon]) and aetiology were extracted from electronic medical records based on the final diagnosis documented by the treating neurologist and the corresponding ICD-10 code. A de novo adjudication using strict TOAST criteria was not performed during this retrospective study.

### Inclusion and exclusion criteria

Patients under the age of 50 years who presented with clinical signs consistent with stroke (e.g., acute onset of focal weakness, speech disturbance, paraesthesia, ataxic gait, visual field defects, etc), confirmed via neuroimaging (non-contrast CT scan of the head), after reasonable exclusion of common stroke mimics (e.g., hypoglycaemia, seizure, etc) were included in the study. Exclusion criteria included traumatic intracranial bleeding, intracranial masses presenting with stroke-like symptoms and those diagnosed with transient ischemic attack. No patients during the study period were excluded based on region of origin, sex or other demographic variables.

### Sample size

A total of 419 patients met the inclusion criteria and were included in the analysis. While no formal minimum sample size calculation was performed due to the retrospective nature of the study, existing literature suggests that sample sizes of 100 patients or more are generally sufficient for summarizing categorical and continuous variables in observational studies. Furthermore, samples in the range of 200–400 charts have been shown to provide reasonably precise estimates, with 95% confidence intervals typically within 5–15% of the mean [11].

### Statistical analysis

Data was imported into python 3 and analyzed using the Matplotlib v3.3.4, pandas v1.2.4, statsmodels v0.12.2, and scipy-v1.10.0, packages. For each variable, appropriate groupings were defined and used based on similarity or previous literature. Missingness was dealt with on a case-by-case basis, and during bivariate analysis, through pairwise deletion. Patient regions of origin were aggregated to the 21 regions as defined by the 2023 Global Burden of Disease framework [12]. For analysis purposes, the regions were further aggregated into four primary categories: MENA, South Asia, Sub-Saharan Africa, and Others. This aggregation aligns with broader regional taxonomies used in multiregional cohorts [13]. Chi-squared tests were used for bivariate analyses given the categorical nature of the variables. P values less than 0.05 were taken to be significant.

## Results

**Fig 1:**
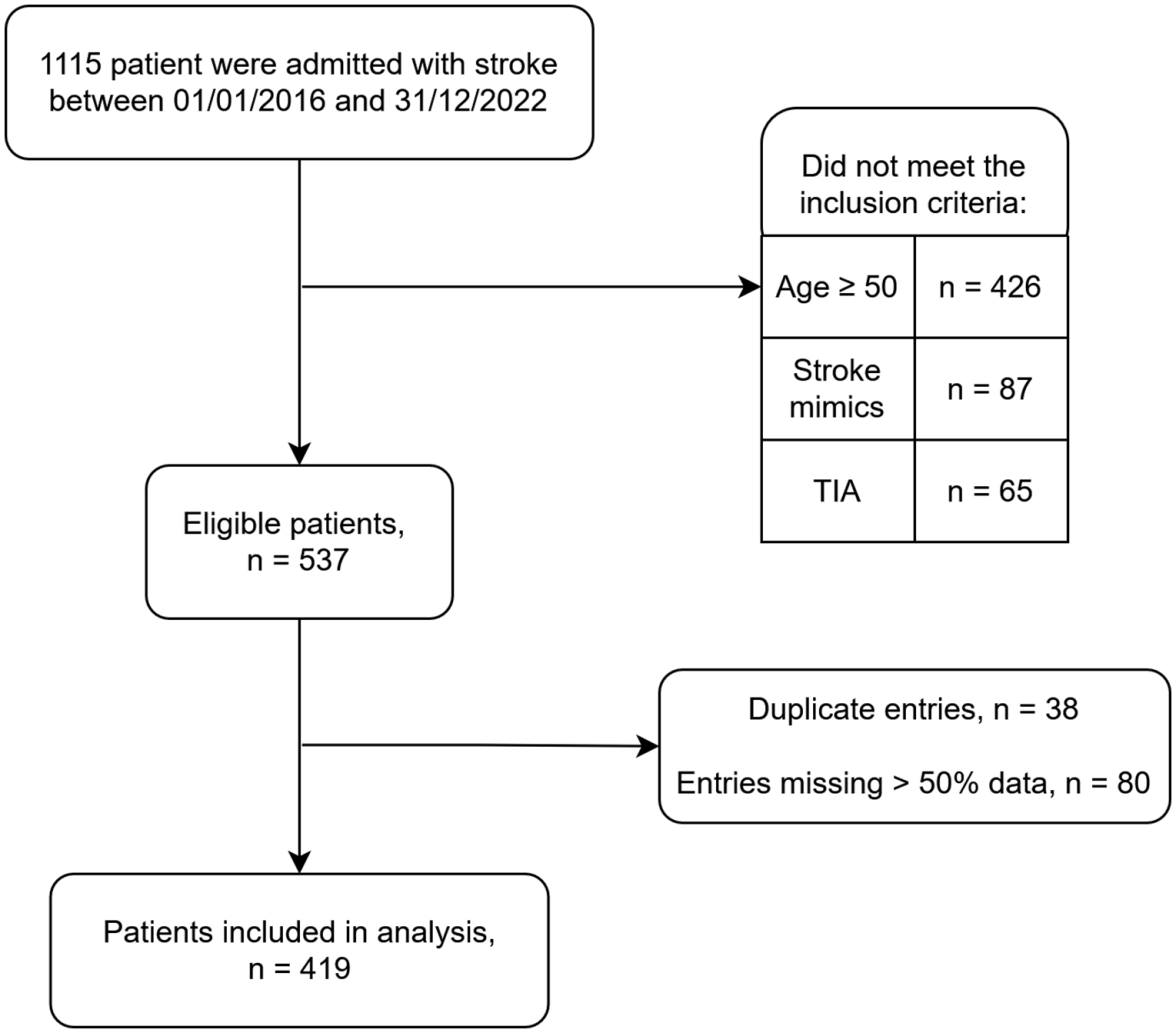
Patient selection flowchart.

A total of 537 cases were initially extracted from the electronic medical records. Duplicate entries (strictly defined as redundant records sharing the same Encounter ID and admission date) and cases with more than 50% missing data were dropped during data cleaning (Fig 1).

As such, 419 patients were included in the study. The majority of patients were of South Asian origin (63.96%, n = 268), followed by patients from the MENA region (19.33%, n = 81), and a smaller proportion from other regions (11.22%, n = 47) and Sub-Saharan Africa (5.49%, n = 23). Most patients were male (78.28%, n = 328), and mean age was 39.99 ± 7.24. Further demographic details are displayed in Table 1.

**Table 1:** Demographic Characteristics of Study Participants.

	Overall stroke	Ischemic stroke	Intracerebral hemorrhage	Non-traumatic subarachnoid hemorrhage
Age	39.99 ± 7.24	38.57 ± 7.58	42.57 ± 6.26	40.03 ± 8.43
BMI	27.26 ± 5.01	27.77 ± 4.86	26.35 ± 4.51	27.68 ± 5.75
Systolic blood pressure	156.80 ± 35.13	157.01 ± 33.93	161.38 ± 40.88	150.88 ± 28.31
Diastolic blood pressure	93.60 ± 22.54	94.49 ± 22.67	96. 24 ± 25.40	88.53 ± 17.55
**Racial/Ethnic group**				
South Asia	63.96% (n = 268/419)	58.58% (n = 157/268)	22.39% (n = 60/268)	19.03% (n = 51/268)
MENA	19.33% (n = 81/419)	37.04% (n = 30/81)	30.86% (n = 25/81)	32.10% (n = 26/81)
Others	11.22% (n = 47/419)	23.40% (n = 11/47)	53.19% (n = 25/47)	23.40% (n = 11/47)
Sub-Saharan Africa	5.49% (n = 23/419)	52.17% (n = 12/23)	17.39% (n = 4/23)	30.43% (n = 7/23)
**Sex**				
Male	78.28% (n = 328/419)	85.24% (n = 179/210)	67.54% (n = 77/114)	75.79% (n = 72/95)
Female	21.72% (n = 91/419)	14.76% (n = 31/210)	32.46% (n = 37/114)	24.21% (n = 23/95)
**Type of stroke**				
Ischemic stroke	50.12% (n = 210/419)	–	–	–
Intracerebral hemorrhage	27.21% (n = 114/419)	–	–	–
Non-traumatic subarachnoid hemorrhage	22.67% (n = 95/419)	–	–	–

MENA: Middle East and North Africa | *Blood pressure data are presented as mean (mmHg) ± SD recorded at the time of admission | Denominators vary due to missing data in electronic records

Regarding stroke type, ischemic stroke was the most prevalent, accounting for 50.12% (n = 210) of cases, followed by intracerebral hemorrhage (27.21%, n = 114) and SAH (22.67%, n = 95). On admission, ECG findings showed that 56.63% (n = 141/249) of patients had a normal sinus rhythm, 18.07% (n = 45/249) had left ventricular hypertrophy, and 25.30% (n = 63/249) presented with other abnormalities.

Only a small proportion of patients had a family history of stroke (2.44%, n = 3/123), and 4.67% (n = 17/364) had a previous personal history of stroke. Atrial fibrillation was observed in only 1.05% (n = 4/380) of patients. Hypertension was present in 45.45% (n = 175/385) of patients, while 24.42% (n = 94/385) had diabetes mellitus, and 7.14% (n = 27/378) had history of coronary heart disease. Among female patients, 1.10% (n = 1/91) reported using oral contraceptives. Most patients (98.71%, n = 384/389) were not on warfarin or heparin prior to admission.

Significant differences in the distribution of the type of stroke were observed based on region of origin (p < 0.001), sex (p < 0.001) and diabetes mellitus status (p = 0.007). Females were 2.23 times more likely to have a hemorrhagic stroke than males (95% CI: 1.37–3.65, p = 0.001). Conversely, patients with ischemic strokes were 2.13 times more likely to have diabetes mellitus as a comorbidity than those with hemorrhagic stroke (95% CI: 1.17–3.88, p = 0.014). Interestingly, hypertension was not significantly associated with hemorrhagic transformation within 24 hours (p = 0.38). Further clinical characteristics are displayed in Table 2.

**Table 2:** Clinical Characteristics of Study Participants.

	Overall stroke	Ischemic stroke	Intracerebral hemorrhage	Non-traumatic subarachnoid hemorrhage
ECG: Left ventricular hypertrophy	18.07% (n = 45/249)	19.88% (n = 33/166)	45.45% (n = 10/22)	3.28% (n = 2/61)
ECG: Normal sinus rhythm	56.63% (n = 141/249)	62.65% (n = 104/166)	27.27% (n = 6/22)	50.82% (n = 31/61)
ECG: Other abnormalities	25.3% (n = 63/249)	17.47% (n = 29/166)	27.27% (n = 6/22)	45.9% (n = 28/61)
Hypertension	45.45% (n = 175/385)	44.83% (n = 91/203)	53.54% (n = 53/99)	37.35% (n = 31/83)
Diabetes mellitus	24.42% (n = 94/385)	30.88% (n = 63/204)	17.35% (n = 17/98)	16.87% (n = 14/83)
Coronary artery disease	7.14% (n = 27/378)	7.0% (n = 14/200)	5.21% (n = 5/96)	9.76% (n = 8/82)
Oral contraceptive use (Females)				
No	98.90% (n = 90/91)	–	–	–
Yes	1.10% (n = 1/91)	–	–	–
TOAST classification of ischemic stroke				
Cardioembolic stroke		3.81% (n = 8/210)	–	–
Large-artery atherosclerosis		24.76% (n = 52/210)	–	–
Small-vessel disease		10.95% (n = 23/210)	–	–
Stroke of other determined aetiology		0% (n = 0/210)	–	–
Stroke of undetermined aetiology		46.19% (n = 97/210)	–	–
IV thrombolytic therapy	9.41% (n = 37/393)	19.79% (n = 37/187)	–	–
Endovascular interventions	0.49% (n = 2/409)	0.48% (n = 1/210)*	0% (n = 0/114)	1.05% (n = 1/95)^
Overall mortality	12.68% (n = 53/418)	7.62% (n = 16/210)	21.93% (n = 25/114)	12.77% (n = 12/94)

ECG: electrocardiogram; IV: intravenous | * mechanical thrombectomy | ^ aneurysmal coiling | Denominators vary due to missing data in electronic records

Cardioembolic stroke was identified in 2.82% (n = 8/284) of cases, and thrombophilia was present in 1.90% (n = 3/158) of patients. Large-artery atherosclerosis was observed in 22.47% (n = 60/267) of cases, and small-vessel disease was identified in 13.99% (n = 48/343). The cause of stroke remained undetermined in 46.78% (n = 196/419) of patients. Regarding prognosis, patients with hemorrhagic strokes were 3.41 times more likely to have in-hospital mortality compared to those with ischemic strokes (95% CI: 1.73–6.69, p < 0.001).

In terms of treatment, intravenous thrombolytic therapy was administered to 9.41% (n = 37/393) of patients, with abciximab being administered with IV tPA in only 0.60% (n = 1/166) of cases. Aspirin was given to 42.24% (n = 177/419) of patients, while mechanical thrombectomy was performed in 0.24% (n = 1/409) of cases. Hemorrhagic transformation occurred in 6.19% (n = 21/339) of patients. The overall mortality rate was 12.68% (n = 53/418). Importantly, those with a history of coronary artery disease had 4.65 times higher mortality rates (p < 0.001).A subgroup univariate analysis comparing South Asian and non-South Asian patients was conducted. Compared to the non-South Asian group, South Asian patients were 4.82 times more likely to receive intravenous thrombolytic therapy (95% CI: 4.22–5.51, p < 0.001) and 2.19 times more likely to present with large-artery atherosclerosis (95% CI: 1.11–4.30, p = 0.02). Interestingly, the overall mortality was comparable between the groups (12.36% in South Asians vs. 13.25% in non-South Asians, p = 0.10). Detailed overview of the comparative outcomes between the two groups is highlighted in Table 3.

**Table 3:** Comparison of baseline characteristics, management, and outcomes of ischemic stroke in South Asian vs non-South Asian patients.

	South Asian	Non-South Asian
IV thrombolytic therapy	13.31% (n = 33/248)	2.76% (n = 4/145)
Aspirin administered	48.51% (n = 130/268)	31.13% (n = 47/151)
Mechanical thrombectomy	0.38% (n = 1/263)	0% (n = 0/146)
Cardioembolic stroke	1.75% (n = 3/171)	4.42% (n = 5/113)
Large-artery atherosclerosis	26.7% (n = 47/176)	14.29% (n = 13/91)
Small-vessel disease	11.57% (n = 25/216)	18.11% (n = 23/127)
Cause of stroke is undetermined	48.51% (n = 130/268)	43.71% (n = 66/151)
Thrombophilia	2.2% (n = 2/91)	1.49% (n = 1/67)
Hemorrhagic transformation within 24 hours	5.16% (n = 11/213)	7.94% (n = 10/126)
Overall mortality	12.36% (n = 33/267)	13.25% (n = 20/151)

IV: intravenous | Denominators vary due to missing data in electronic records

## Discussion

This study offers significant insights into the demographic and clinical characteristics of young-onset stroke patients in the UAE, with a particular focus on risk factors, outcomes, and the unique demographic composition of those patients.

In this study the majority of patients with young-onset stroke were from South Asia, followed by the MENA region; this reflects the UAE's racial/ethnic composition, with South Asian people contributing to 59.4% of the population [14]. In contrast, studies conducted in other MENA countries, such as Saudi Arabia and Kuwait, typically involve a larger proportion of native populations. In one particular Saudi study, most of the stroke patients were native Saudis [15]. Similar to our findings, males made up the majority of the stroke cohort in the MENA region, reflecting a higher stroke incidence among males, which has also been documented globally [16].

Data on the unique characteristics of young-onset stroke patients is limited. In our cohort, ischemic stroke was the most common type, accounting for about half of the cases, while intracerebral hemorrhage and SAH each comprised approximately a quarter. These findings contrast with a large-scale study conducted in the MENA region, which reported 13.9% of stroke cases as hemorrhagic and only 1.0% as SAH [16]. The higher prevalence of SAH in our cohort reflects reported epidemiological differences of young-onset stroke; hemorrhagic stroke frequently comprise a larger proportion in young individuals when compared to older individuals [17]. Furthermore, while our centre serves as the primary stroke referral facility for the Emirate of Sharjah, it is not a specialized neurosurgical hub for complex aneurysm interventions; thus, this distribution suggests a genuine demographic trend rather than a surgical selection bias. Given that hypertension is a dominant risk factor for hemorrhagic strokes, and intracerebral hemorrhage being strongly associated with diabetes mellitus, these results highlight the need for more aggressive prevention and management strategies targeting modifiable risk factors in the UAE.

When compared to data from Western nations, like the United States (US) and European nations, some key differences emerge. In our study, the prevalence of hypertension (45%) and diabetes mellitus (24%) was notably higher than that observed in stroke cohorts in Western nations. For example, studies from the US report hypertension in about 35% of stroke patients, with diabetes mellitus affecting approximately 20% of cases [18]. This suggests that stroke patients in the UAE may have a heavier burden of these comorbidities. Additionally, the male predominance (78%) in our study is more pronounced than in studies from the West, where sex disparities are less stark, potentially reflecting regional lifestyle and occupational differences [1,19].

It is important to interpret the observed associations between region of origin and stroke subtype within the socioeconomic context of the UAE. For instance, region of origin frequently correlates with occupational status and insurance tiers. In our study, South Asian patients were disproportionately affected by young-onset stroke and had a significantly higher prevalence of large-artery atherosclerosis. While the exact cause of this finding remains unclear, it is likely the result of multiple interrelated factors. The South Asian population in the UAE is largely composed of male manual labourers, who may be exposed to specific environmental stressors and dietary patterns distinct from other groups. A recent multiethnic study from the region suggested that this population faces a ‘double burden’ of risk. This likely involves an interplay between genetic predispositions (i.e., genetic polymorphism, increased susceptibility to metabolic syndrome and smaller arterial diameters) and unique environmental stressors inherent to the migrant labour experience [20]. Furthermore, several lifestyle risk factors are prevalent in this population, including hypertension, diabetes mellitus, tobacco and alcohol use, and sedentary lifestyle [21]. Conversely, disparities in health-seeking behaviour are often driven by cultural perceptions of illness or financial barriers which may influence presentation time. This is particularly relevant for those with lower insurance coverage, who may present later or less frequently for mild deficits [22], potentially skewing the observed demographic and severity distributions in our cohort.

Importantly, in our study we observed a relatively high proportion (~45%) of strokes of undetermined aetiology. This could be due to the cardiac monitoring being limited to inpatient telemetry and TTE, and a lack of utilisation of outpatient Holter monitor or implantable loop recorders. Multiple trials have demonstrated that prolonged monitoring (i.e., 30-day monitoring or implantable loop recorders) are more likely to detect atrial fibrillation [23,24]. Additionally, transoesophageal echocardiography and TTE with bubble or contrast studies are not regularly performed at our centre. Hence, the underutilisation of such monitoring devices and advanced echocardiographic imaging might be artificially inflating the “strokes of undetermined aetiology” in this study.

A critical area for improvement in the UAE, as highlighted by our study, is the very low rate of mechanical thrombectomy. In contrast, mechanical thrombectomy rates in the US and Europe are significantly higher, often exceeding 10–15% in ischemic stroke patients [19,25]. This gap in access to advanced stroke care highlights the need for improvements in the stroke referral and treatment pathways in the UAE. Given that mechanical thrombectomy has been shown to dramatically improve outcomes for patients with large-vessel occlusions, increasing the availability and uptake of this intervention could significantly reduce mortality and disability rates. Despite the UAE’s status as a high-income country, the underutilisation of acute reperfusion therapy in our study is attributed to infrastructural and workforce limitations rather than financial barriers. In our tertiary care centre, the absence of a dedicated 24/7 neuro-interventional service and the reliance on a single interventional radiologist significantly hinders access to mechanical thrombectomy. This highlights a critical gap in subspecialty coverage, suggesting that financial resources alone are insufficient without parallel investment in healthcare workforce development. Addressing this issue is imperative to improving access to advanced stroke care and outcomes. Furthermore, the high proportion of hemorrhagic strokes reduces the pool of eligible patients for reperfusion therapy when compared to an older cohort with a higher burden of large-vessel occlusion.

Worldwide, the overall age-standardised mortality rate of stroke decreased by 36.0% between 1990 and 2019 [1]. Similarly, a US study showed a consistent decline in in-hospital mortality rates over the years, reaching just 2.1% in 2017 [26]. However, given that our study focuses on young-onset stroke patients, an in-hospital mortality rate of 12.68% is especially elevated. Such striking differences in mortality rates could be attributed to the low availability of advanced interventions and the higher prevalence of comorbid conditions in our cohort. The higher mortality among younger stroke patients in the UAE compared to international populations underscores the importance of optimising acute stroke care and improving the management of stroke risk factors.

### Strengths

One of the primary strengths of this study is its focus on young-onset stroke, an area that has been underexplored in the MENA region. Regional studies, including those conducted in Qatar, Saudi Arabia and the MENA region, have mainly focused on general age groups without stratifying for young-onset stroke, lacked analysis per region of origin, or had a limited sample size. The relatively large sample size of 419 patients allows for robust statistical analysis and provides a comprehensive picture of stroke characteristics in the UAE. Additionally, by capturing both native and expatriate populations, our study reveals that South Asian patients are disproportionately affected compared to other groups, a key public health finding for the Gulf region. Additionally, the use of standardised diagnostic codes (ICD-10) and detailed clinical records strengthens the reliability of the data collected.

### Limitations

Notably, this study has several limitations inherent to its retrospective design. Firstly, as the primary stroke referral centre in the Emirate, our findings may be subject to selection bias particularly regarding the high proportion of SAH. Secondly, the initial data query relied on ICD-10 coding without demographic- and region-specific validation. While a recent study validated specific ICD-10 codes to maximize incident stroke capture in an older US population [27], these codes remain unvalidated in younger patients in the MENA region. Additionally, the study relies heavily on the completeness and accuracy of the electronic medical records and ICD coding, this has resulted in missing data for some variables and inability to reliably extract some variables such as the door-to-needle times. Moreover, some important lifestyle factors, such as smoking and alcohol use, may not have been consistently recorded, potentially underestimating their role in stroke risk. Thirdly, we could not determine the exact number of patients with large vessel occlusion who were eligible for mechanical thrombectomy but did not receive it. Retrospectively correlating the precise presentation times of these patients with the historical, specific availability of the single interventional radiologist was not logistically feasible, limiting our ability to quantify the exact missed opportunity rate. Fourthly, the study does not provide long-term follow-up data, limiting the ability to assess functional recovery, disability, or recurrent stroke events; our functional outcomes were limited to either in-hospital mortality or survival to discharge. Finally, the low rate of mechanical thrombectomy utilisation restricts the generalisability of the results to centres with more developed stroke units.

## Conclusion

This study provides valuable insights into the epidemiology of young-onset stroke in the UAE, with a particular focus on modifiable risk factors such as hypertension and diabetes mellitus. Compared to both regional and international data, our findings highlight several critical areas for improvement in stroke care, particularly in the early management of ischemic stroke and the utilisation of advanced treatment modalities. Future research should aim to explore the long-term outcomes of young-onset stroke patients in the UAE, as well as identify additional risk factors that may be unique to this population. Strengthening public health interventions focused on reducing hypertension and diabetes mellitus, as well as improving access to stroke care, will be essential in mitigating the burden of stroke in younger adults.

### Strengths and limitations of this study

1. The relatively large sample size of 419 patients provides a comprehensive picture of young-onset stroke characteristics in the UAE.
2. The regional diversity of the cohort makes the findings particularly relevant for the UAE’s unique demographic profile.
3. This retrospective study relies on electronic medical and health records as its data source, which may be limited by coding errors or incomplete record.
4. The study does not provide long-term follow-up data, limiting the ability to assess functional recovery, disability, or recurrent stroke events.
5. The low rate of advanced interventions, such as mechanical thrombectomy, may limit the generalizability of the results to regions with more developed stroke systems of care.

## Supporting information

S1 File Supplementary Table 1. ICD-10 Stroke Codes. (DOCX)
