# Supplementary material for: Young-onset stroke in the UAE: A single-centre retrospective analysis of risk factors and outcomes
Source: PLoS One. 2026 Aug 6;21(8):e0355300. doi: 10.1371/journal.pone.0355300 (PMC13446658; doi:10.1371/journal.pone.0355300)
Supplement: Supplementary file 1 [file pone.0355300.s001.docx]

| **Category** | **ICD-10 Code** | **Description** |
| --- | --- | --- |
| **Subarachnoid Hemorrhage** | **I60.x** | **Nontraumatic subarachnoid hemorrhage** |
|  | I60.00 | From unspecified carotid siphon and bifurcation |
|  | I60.01 | From right carotid siphon and bifurcation |
|  | I60.02 | From left carotid siphon and bifurcation |
|  | I60.10 | From unspecified middle cerebral artery |
|  | I60.11 | From right middle cerebral artery |
|  | I60.12 | From left middle cerebral artery |
|  | I60.2 | From unspecified anterior communicating artery |
|  | I60.30 | From unspecified posterior communicating artery |
|  | I60.31 | From right posterior communicating artery |
|  | I60.32 | From left posterior communicating artery |
|  | I60.4 | From basilar artery |
|  | I60.50 | From unspecified vertebral artery |
|  | I60.51 | From right vertebral artery |
|  | I60.52 | From left vertebral artery |
|  | I60.6 | From other intracranial arteries |
|  | I60.7 | From unspecified intracranial artery |
|  | I60.8 | Other |
|  | I60.9 | Unspecified |
| **Intracerebral hemorrhage** | **I61.x** | **Nontraumatic intracerebral hemorrhage** |
|  | I61.0 | In hemisphere, subcortical |
|  | I61.1 | In hemisphere, cortical |
|  | I61.2 | In hemisphere, unspecified |
|  | I61.3 | In brain stem |
|  | I61.4 | In cerebellum |
|  | I61.5 | Intraventricular |
|  | I61.6 | Multiple localized |
|  | I61.8 | Other |
|  | I61.9 | Unspecified |
| **Cerebral infarction** | **I63.x** | **Cerebral infarction** |
|  | I63.0 | Due to thrombosis of precerebral arteries |
|  | I63.1 | Due to embolism of precerebral arteries |
|  | I63.2 | Due to unspecified occlusion or stenosis of precerebral arteries |
|  | I63.3 | Due to thrombosis of cerebral arteries |
|  | I63.4 | Due to embolism of cerebral arteries |
|  | I63.5 | Due to unspecified occlusion or stenosis of cerebral arteries |
|  | I63.6 | Due to cerebral venous thrombosis, nonpyogenic |
|  | I63.8 | Other |
|  | I63.9 | Unspecified |
